# Characterization of pregnancy outcome of women with an offspring with inborn errors of metabolism: A population-based study

**DOI:** 10.3389/fgene.2022.1030361

**Published:** 2022-11-09

**Authors:** Tali Epstein Weiss, Offer Erez, Itai Hazan, Amit-Shira Babiev, Orna Staretz Chacham

**Affiliations:** ^1^ Joyce and Irving Goldman Medical School, Faculty of Health Sciences, Ben-Gurion University of the Negev, Beer Sheva, Israel; ^2^ Internal Medicine B, Tel Aviv Ichilov-Sourasky Medical Center, Tel Aviv, Israel; ^3^ Department of Obstetrics and Gynecology, Emek Medical Center, Afula, Israel; ^4^ Department of Obstetrics and Gynecology, Hutzel Women’s Hospital, Wayne State University, Detroit, MI, United States; ^5^ Clinical Research Center, Soroka University Medical Center, Faculty of Health Sciences, Ben Gurion University of the Negev, Beer Sheva, Israel; ^6^ Metabolic Clinic, Soroka University Medical Center, Faculty of Health Sciences, Ben Gurion University of the Negev, Beer Sheva, Israel

**Keywords:** fetal growth restriction, HELLP syndrome, inborn errors of metabolism, neonatal death, nonimmune hydrops fetalis, polyhydramnios, pregnancy characteristics, preterm birth

## Abstract

**Introduction:** Inborn errors of metabolism (IEM) are scarce, and their diagnosis is often made after birth. This has led to the perception that most fetuses affected by these disorders do not become clinically apparent during pregnancy**.** Our aim was to determine the obstetrical characteristics of women with an offspring affected by IEM.

**Methods:** This population-based retrospective cohort study included all women who delivered at the Soroka University Medical Center (SUMC) from 1988 to 2017 who met the inclusion criteria. Mothers who had an offspring with IEM were included in the study group, and those who had offsprings without IEM comprised the comparison group.

**Results:** A total of 388,813 pregnancies were included in the study, and 184 of them were complicated by a fetus with IEM. The number of Bedouin women was higher in the IEM-affected infant group than in the comparison group (90.8% *vs*. 53.3%, *p* < 0.001); women who had a fetus with IEM had a higher rate of polyhydramnios (7.1% *vs*. 3.2%, *p* = 0.005), HELLP syndrome (3.3% *vs*. 1.1%, *p* = 0.014), and preterm birth (20.7% *vs*. 10.1%, *p* < 0.001); neonates with IEM had lower mean birth weight (*p* < 0.001), lower Apgar scores at 1′ and 5′ minutes (*p* < 0.001), and a higher rate of fetal growth restriction (FGR) (*p* < 0.001), postpartum death <28 days (*p* < 0.001), and neonatal death (*p* < 0.001) than those in the comparison group. Pregnancies with IEM fetuses were independently associated with preterm birth (OR 2.00; CI 1.4–3), polyhydramnios (OR 2.08; CI 1.17–3.71), and FGR (OR 2.24; CI 1.2–4.19). Each family of metabolic diseases is independently associated with specific pregnancy complications (i.e., mitochondrial diseases are associated with HELLP syndrome (OR 5.6; CI 1.8–17), and lysosomal storage disease are associated with nonimmune hydrops fetalis (OR 26.4; CI 3.39–206).

**Conclusion:** This study reports for the first time, an independent association of IEM with specific complications of pregnancy. This observation has clinical implications, as the identification of specific pregnancy complications in a population at risk for IEM can assist in the prenatal diagnosis of an affected fetus.

## Introduction

Inborn errors of metabolism (IEM) are rare diseases that can be clinically presented and diagnosed in fetuses, newborns (through newborn screening tests) (Pourfarzam and Zadhoush, 2013; Kliegman et al., 2016), or later on in life, even in adulthood. IEM affect 1/800–2,500 deliveries worldwide (Behrman richard and Klieegman robert, 2018). Its prevalence reaches from 56.6 to 100,000 or 1/2000 live births among the Bedouin population (Hazan et al., 2020) that resides in the Negev region of the southern part of Israel and has a high consanguinity rate (Singer et al., 2020). Indeed, the incidence of glycogen storage diseases (GSD) among the Bedouins is 11.2 in 100,000 or 1.12/10,000 live births (Hazan et al., 2020) when compared to the 1/20,000–40,000 live births in Europe, Canada, and the United States (Pritchard and Center, 2019), thus giving us a unique opportunity to study the prenatal characteristics of IEM in our population.

The current perception is that in most cases, the clinical features of IEM become apparent after delivery probably because most of the metabolic toxic products that cannot be metabolized by the fetus are secreted by the trophoblast and removed by the mother, who acts as a human filter (Zand et al., 2003; Illsinger and Das, 2010). Although the mother serves as the “detoxifier” of the fetus, there are reports of antenatal clinical signs in the fetus. For example, nonimmune hydrops fetalis (NIHF) can be the clinical presentation of fetal lysosomal storage disorders (LSD) (Stone and Sidransky, 1999; Staretz-Chacham et al., 2009) [e.g., type 2 Gaucher disease (Sudrié-Arnaud et al., 2018), galactosialidosis (Haverkamp et al., 1996), Niemann–Pick disease type C (Meizner et al., 1990; Spiegel et al., 2009; Surmeli-Onay et al., 2013)], GSD type IV (Alegria et al., 1999) and 1a (Edwards et al., 2006), and peroxisomal diseases (e.g., Zellweger disease) (Dursun et al., 2009). As described by Spiegel et al. (2009), the prenatal presentation of Niemann–Pick disease type C in a fetus could also be splenomegaly, hepatomegaly, or ascites as part of NIHF and less common as fetal growth restriction (FGR), oligohydramnios, and placentomegaly (Spiegel et al., 2009). Additionally, fetuses affected with nonketotic hyperglycinemia (NKH) may suffer from hiccups and seizures (Ambani et al., 1979). Moreover, fetal metabolic waste products can affect the mother. Indeed, fetuses with long-chain L-3 hydroxyacyl-CoA dehydrogenase deficiency (LCHAD) (Wilcken et al., 1993; Preece and Green, 2002; Journal et al., 2005) and other fetal fatty acid oxidation defects (FAOD) (Innes et al., 2000; Matern et al., 2001; Nelson and Alters, 2018) may cause maternal HELLP (hemolysis, elevated liver enzymes, and low platelets) syndrome or acute fatty liver of pregnancy (AFLP) of the mother.

The prenatal diagnosis of gestations complicated by IEM is important, as some of the mothers and the affected fetuses will benefit from specialized maternal and fetal care. For example, fetuses affected by cobalamin disorders will benefit from maternal supplementation of vitamin B12 and biotin in biotinidase deficiency (Walter, 2000; Hayek et al., 2019) or galactose-free diet for a mother carrying a fetus affected by galactosemia (Illsinger and Das, 2010), suggesting that there is a need to determine the clinical features during pregnancy that are associated with the fetus presenting with IEM in the population at risk. Therefore, the high prevalence of IEM in our population allows us to construct a unique and one of the largest cohorts of IEM-affected pregnancies, along with the long-term follow-up of the neonates in a specialized metabolic pediatric clinic, thereby allowing us to address this question. Thus, we conducted a population-based retrospective cohort study that aimed to determine the obstetrical characteristics of women with an offspring affected by IEM as a general disorder and stratified it according to the different families of IEM.

## Material and methods

### Study and population

This population-based retrospective cohort study included all women who delivered at the Soroka University Medical Center (SUMC) from 1988 to 2017. The demographic and medical information of the patients included in the cohort was retrieved by the SUMC computerized database, capturing the patient’s medical records and diagnoses at hospitalization coded according to the International Classification of Disease 9th revision (ICD-9). The ICD-9 codes of the IEM are as follows: fatty acid oxidation disease (FAOD) 277.85; glutaric aciduria (GA) 270.7, 277.85, and 277.86; glycogen storage diseases (GSD) 271; mitochondrial diseases 277.87; mucopolysaccharidoses (MPS) 277.5; maple syrup urine disease (MSUD) 270.3; nonketotic hyperglycinemia (NKH) 790.29; and Niemann–Pick disease 272.7. Women who had fetuses with congenital malformations or with chromosomal abnormalities not associated with IEM were excluded from the study. The study was approved by the SUMC Institutional Review Board.

### Clinical definitions

Hypertension is defined as blood pressure ≥140/90 mmHg recorded in two separate measurements at least 4 h apart. Mild hypertension is defined as a diastolic blood pressure ≥90 mmHg and <110 mmHg and/or systolic blood pressure ≥140 mmHg and <160 mmHg. Severe hypertension is defined as the presence of diastolic blood pressure ≥110 and systolic blood pressure ≥160. Gestational hypertension is defined as the presence of hypertension developed after 20 weeks of gestation without proteinuria. HELLP syndrome is diagnosed as the detection of all three laboratory abnormalities comprising hemolysis [anemia—hemoglobin level <8–10 g/dl (depending on the trimester), serum bilirubin ≥1.2 mg/dl, and low serum haptoglobin ≤25 mg/dl or lactate dehydrogenase (LDH) ≥2 times the upper level of the normal] with a microangiopathic blood smear (fragmented red blood cells, i.e., schistocytes and burr cells); elevated liver enzymes—aspartate aminotransferase (AST) or alanine aminotransferase (ALT) ≥2 times the upper level of the normal; and low platelet count <100,000 cells/μL in a pregnant/postpartum woman. Preeclampsia was diagnosed in the presence of elevated blood pressure and proteinuria of at least +1 in dipstick; its severity is defined according to the severity of hypertension and/or one of the following: +3 proteinuria by dipstick, thrombocytopenia ≤100,000, elevated liver enzymes, persistent headache, and/or blurred vision. Gestational diabetes is diagnosed according to the oral glucose tolerance test and classified according to White’s classification. While hydramnios is when the amniotic fluid index (AFI) >25 cm or when a vertical pocket of at least 8 cm was measured or as a subjective estimation of increased amniotic fluid volume and oligohydramnios is when the AFI <5 cm; a real-time scanner equipped with a 3.5/5 MHz transducer of appropriate focal length estimated amniotic fluid volume. Preterm delivery is defined as delivery before completing 37 weeks of gestation. NIHF is defined as abnormal fluid accumulation in at least two fetal serous cavities (e.g., ascites, pleural effusions, and/or pericardial effusions), often accompanied by skin edema, that does not result from red cell alloimmunization (e.g., RhD and Kell).

Newborns were classified according to their weight as follows: small for gestational age (SGA)—birthweight less than the 10th percentile, appropriate for gestational age (AGA)—birthweight from 10 to 90th percentile, and large for gestational age (LGA)—birthweight more than the 90th percentile according to regional growth curves. Premature rupture of membranes (PROM) was defined as rupture of the chorioamniotic membranes before the onset of labor. Postpartum fever—maternal temperature ≥38°C that developed at least 24 h after delivery, recorded in two different measurements at least 4 hours apart or as one measurement of maternal temperature of ≥38.5°C regardless of the time after delivery. Endometritis—postpartum maternal fever with clinical signs of tenderness above the uterine fundus or during cervical manipulation, foul vaginal discharge, and positive endometrial culture. Wound infection was defined according to either clinical signs of infection or positive wound culture. Wound dehiscence—spontaneous opening of cesarean section wound that included the abdominal fascia.

### Statistical analysis

Continuous variables are reported as mean ± SD for normal distribution or as median with interquartile range (IQR) for non-normal distribution. Categorical variables are presented as percentages. Comparisons were made using appropriate statistical tests. Continuous variables were compared using an unpaired t-test, ordinal variables were compared using the Mann–Whitney U-test, and nominal variables were compared using chi-square or Fisher’s exact test.

### Multivariate analysis

We conducted a generalized estimating equation (GEE) with a logistic distribution. The unit of analysis was the fetuses. Fetuses diagnosed with IEM were defined as the dependent variable, the mother as the random variable, and pregnancy complications as the primary independent variables. The regression was adjusted for polyhydramnios, ethnicity, HELLP syndrome, FGR, and preterm delivery, which differed significantly between the study groups in the univariate analysis. The results of the GEE models are presented as an odds ratio (OR), 95% confidence interval (CI), and *p*-value. A two-sided *p*-value <0.05 was considered statistically significant for all statistical tests; *p*-values reported were rounded to two decimal places. All statistical analyses were performed using the R and SPSS statistical software.

## Results

### Epidemiological characteristics

The IEM group included mothers who had an affected offspring (*n* = 184) and the comparison group included mothers who had offsprings without IEM (*n* = 338,629). Maternal demographics and pregnancy characteristics are described in Table 1. The mean maternal age was lower in the IEM group in relation to the comparison group (*p* = 0.004). The number of Bedouin women was higher in the IEM group than in the comparison group [167 (90.8%) *vs*. 180,560 (53.3%), *p* < 0.001]. The median social state score was lower in the IEM group than in the comparison group [2.00 (0.00,3.00) *vs*. 3.00 (0.00, 9.00), *p* < 0.001]. There were more recurrent pregnancy losses in the IEM group than in the comparison group (10.9% *vs*. 6.8%, *p* = 0.038).

**TABLE 1:** Maternal demographic and pregnancy characteristics.

Characteristics	Comparison group (*n* = 338,629)	Inborn errors of metabolism (*n* = 184)	*p*-value
Maternal age (years)	28.80 ± 5.84	27.55 ± 5.55	**0.004**
Bedouin origin	180,560 (53.3)	167 (90.8)	**<0.001**
Social state score	3.0 [0.0, 9.0]	2.0 [0.0, 3.0]	**<0.001**
Married	138,534 (40.9)	113 (61.4)	**<0.001**
Gravidity	3.0 [2.0, 5.0]	4.0 [2.0, 6.0]	**0.003**
Parity	3.0 [2.0, 4.0]	3.0 [2.0, 5.0]	**0.005**
Infertility treatment	10,230 (3.0)	1 (0.5)	0.08
Recurrent pregnancy loss	22,866 (6.8)	20 (10.9)	0.038
Diabetes mellitus	4,362 (1.3)	1 (0.5)	0.570
Hypertension	196 (0.1)	0 (0.0)	1.000
Obesity	1,250 (0.4)	0 (0.0)	0.828
Smoking	5,171 (10.2)	1 (1.9)	0.081
Alcohol use	251 (0.2)	0 (0.0)	1

Data are presented as n (%); mean (S.D.); median [interquartile range].

### Characteristics of inborn errors of metabolism group

**FIGURE 1:**
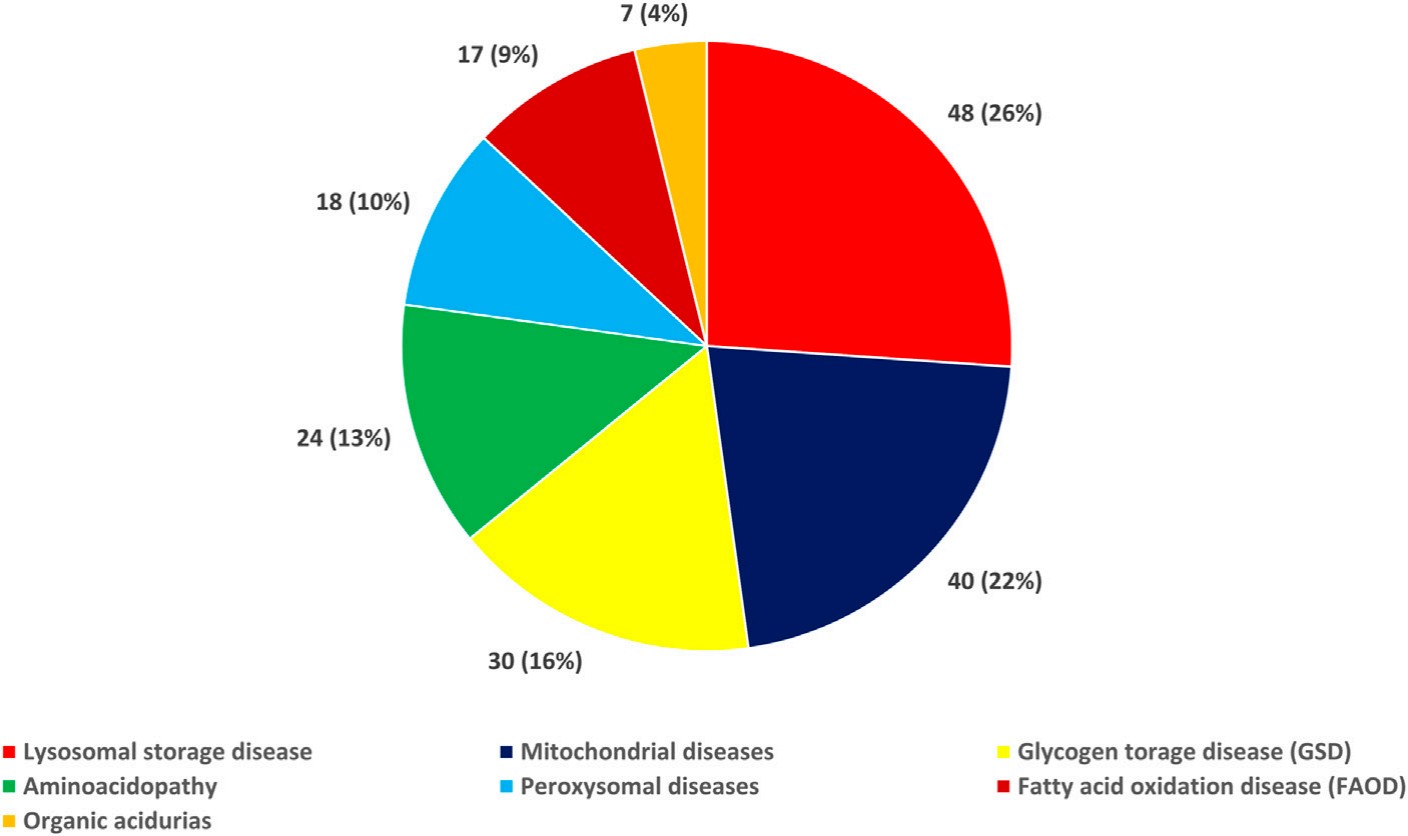
Distribution of the types of inborn errors of metabolism in the study population.

Figure 1 (and Supplementary Table S1) presents the distribution of the IEM types in the study population. As a group, LSD was the most common type of IEM [26.1% (48/184)], followed by mitochondrial diseases [21.7% (40/184)], glycogen storage disease (GSD) [16.3% (30/184)], aminoacidopathy [13.0% (24/184)], peroxisomal diseases [9.8% (18/184)], FAOD [9.2% (17/184)], and organic acidurias [3.8% (7/184)]. We also found that the incidence of LSD was 14.1 per 100,000, while that of mitochondrial diseases was 11.8 per 100,000 and of GSD was 8.8 per 100,000.

### Pregnancy outcome

The pregnancy outcomes are described in Table 2. Women carrying a fetus with IEM had a higher rate of polyhydramnios (*p* = 0.005), preterm deliveries (*p* < 0.001), HELLP syndrome (*p* = 0.014), and NIHF (*p* < 0.001) than those in the comparison group. Moreover, hospitalization length (*p* < 0.001) differed significantly between the study groups.

**TABLE 2:** Pregnancy outcome.

	Outcome	Comparison group (*n* = 338629)	Inborn errors of metabolism (*n* = 184)	*p*-value
Antepartum	Polyhydramnios	10,771 (3.2)	13 (7.1)	**0.005**
	Oligohydramnios	8,064 (2.4)	6 (3.3)	0.589
	Gestational diabetes	13,699 (4.0)	7 (3.8)	1.000
	Gestational hypertension	4,485 (1.3)	3 (1.6)	0.968
	Preeclampsia	14,336 (4.2)	11 (6.0)	0.321
	HELLP syndrome^a^	3,714 (1.1)	6 (3.3)	**0.014**
	Acute fatty liver of pregnancy	94 (0.0)	0 (0.0)	1.000
	Nonimmune hydrops fetalis	74 (0.0)	2 (1.1)	**<0.001**
Intrapartum	Preterm delivery	34,124 (10.1)	38 (20.7)	**<0.001**
	Preterm PROM^b^	36,393 (10.7)	23 (12.5)	0.517
	Mode of delivery			**<0.001**
	Vaginal delivery	267,168 (78.9)	136 (73.9)	
	Caesarean section	55,144 (16.3)	32 (17.4)	
	Breech delivery	616 (0.2)	2 (1.1)	
	Vacuum delivery	10,910 (3.2)	5 (2.7)	
	Hospitalization days	4.00 [3.00, 5.00]	4.00 [3.00, 6.00]	**<0.001**
	ICU^c^ admission	707 (0.2)	1 (0.6)	0.834
Postpartum	Endometritis	9 (0.0)	0 (0.0)	1.000
	Urinary tract infection	1,148 (0.3)	2 (1.1)	0.267
	Infection	2,679 (0.8)	1 (0.5)	1.000
	Fever	1,042 (0.3)	1 (0.5)	**0.002**

Data are presented as n (%); mean (S.D.); median [interquartile range].

^a^ HELLP: hemolysis, elevated liver enzymes, and low platelet count.

^b^ PROM: premature rapture of membranes.

^c^ ICU: intensive care unit.

### Fetal and neonatal outcome

The mean gestational age at delivery and birthweight was lower in the IEM group than in the control group (*p* = 0.001 and *p* < 0.001, respectively), as were the mean Apgar scores at 1′ and 5′ minutes. The rate of FGR (*p* < 0.001), birth asphyxia (*p* = 0.001), and SGA neonates (*p* = 0.001) were higher in the IEM group. Neonates with IEM had a longer mean hospitalization length and a higher rate of neonatal intensive care unit (NICU) admission (*p* < 0.001), persistent hypoglycemia (*p* < 0.001), lactic acidosis (*p* < 0.001), hyperammonemia (*p* < 0.001), hypotonia (*p* < 0.001), developmental delay (*p* < 0.001), dilated/hypertrophic cardiomyopathy (*p* < 0.001), seizures (*p* < 0.001), postpartum death <28 days (*p* < 0.001), and neonatal death between 29 days and 1 year (*p* < 0.001) than those of the comparison group (Table 3).

**TABLE 3:** Fetal and neonatal outcome.

Outcome	Comparison group (*n* = 338,629)	Inborn errors of metabolism (*n* = 184)	*p*-value
Gender			0.518
Male	173,567 (51.3)	102 (55.4)	
Female	164,993 (48.7)	82 (44.6)	
Gestational age at delivery (weeks)	38.97 ± 1.61	38.1 ± 1.89	**0.001**
Apgar score 1 min < 5	5,847 (1.7)	7 (3.8)	**<0.001**
Apgar score 5 min < 7	2,416 (0.7)	7 (3.8)	**<0.001**
Birthweight (grams)	3,157.14 ± 565.47	2,920.61 ± 662.09	**<0.001**
AGA^a^	291,950 (86.2)	145 (78.8)	**0.001**
LGA^b^	27,240 (8.0)	12 (6.5)	
SGA^c^	16,954 (5.0)	20 (10.9)	
Neonatal ICU^d^ admission	42,702 (12.6)	92 (50.0)	**<0.001**
Hospitalization days	3.0 [1.0, 680.0]	4.0 [2.0.112]	**<0.001**
Fetal growth restriction	7,966 (2.4)	13 (7.1)	**<0.001**
Splenomegaly	4 (0.0)	0 (0.0)	1.000
Hepatomegaly	8 (0.0)	0 (0.0)	1.000
Ascites	14 (0.0)	0 (0.0)	1.000
Antepartum death	1,107 (0.3)	1 (0.5)	1.000
Asphyxia	310 (0.1)	2 (1.1)	**0.001**
Meconium aspiration	118 (0.0)	0 (0.0)	1.000
Hemorrhage	1,227 (0.4)	2 (1.1)	0.307
Shoulder dystocia	473 (0.1)	1 (0.5)	0.632
Persistent hypoglycemia	3,396 (1.0)	18 (9.8)	**<0.001**
Metabolic lactic acidosis	392 (0.1)	3 (1.6)	**<0.001**
Hyperammonemia	9 (0.0)	1 (0.5)	**<0.001**
Hypotonia	13,199 (3.9)	30 (16.3)	**<0.001**
Developmental delay	17,437 (5.1)	43 (23.4)	**<0.001**
Dilated/hypertrophic cardiomyopathy	293 (0.1)	17 (9.2)	**<0.001**
Seizures	13 (0.0)	1 (0.5)	**<0.001**
Postpartum death (<28 days)	2,559 (0.8)	22 (12.0)	**<0.001**
Neonatal death (between 29 days and 1 year)	987 (0.3)	27 (14.7)	**<0.001**

Data are presented as n (%); mean (S.D.); median [interquartile range].

^a^ AGA: appropriate for gestational age.

^b^ LGA: large for gestational age.

^c^ SGA: small for gestational age.

^d^ ICU: intensive care unit.

**FIGURE 2:**
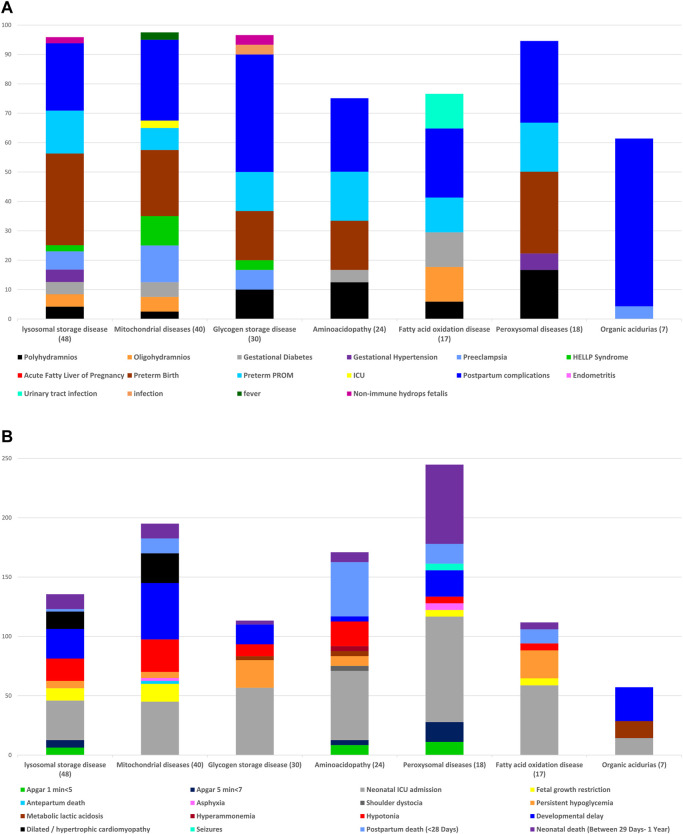
**(A)** Frequency of maternal complications, according to the types of inborn errors of metabolism. **(B)** Frequency of fetal and neonatal outcomes, according to the types of inborn errors of metabolism.

Figures 2A and B (Supplementary Tables S2 and S3) present maternal and neonatal outcomes according to the type of IEM. There are no significant differences among the IEM subgroups in the rate of pregnancy complications, the rate of low Apgar scores at 1′ and 5′ minutes’, the mean birth weight, and length of hospitalization along with the rate of NICU admissions, persistent hypoglycemia, developmental delay, dilated/ hypertrophic cardiomyopathy, and postpartum and neonatal death.

### Multivariate analysis

Polyhydramnios (OR 2.05; CI 1.14–3.67), FGR (OR 2.36; CI 1.28–4.35), preterm delivery (OR 1.93; CI 1.3–2.87), Bedouin origin (OR 8.42; CI 4.92–14.4), and NIHF (OR 13.9; CI 3.16–61.2) were independent risk factors associated with pregnancy complicated with a fetus with IEM (Table 4). The association between clinical risk factors and the delivery of a neonate with IEM is described in Table 5. Pregnancy complications with fetuses with IEM were independently associated with preterm birth (OR 2.00; CI 1.4–3), polyhydramnios (OR 2.08; CI 1.17–3.71), and FGR (OR 2.24; CI 1.2–4.19) but not with HELLP syndrome. The association between fetuses with IEM and pregnancy complications is described in Table 6. The following clinical risk factors were independently associated with LSD: preterm delivery (OR 3.41; CI 1.61–7.22), FGR (OR 3.17; CI 1.06–9.49), Bedouin origin (OR 39.7; CI 5.38–292), and NIHF (OR 26.4; CI 3.39–206). Mitochondrial diseases were independently associated with the following clinical risk factors: FGR (OR 4.7; CI 1.9–12), HELLP syndrome (OR 5.6; CI 1.8–17.0), and Bedouin origin (OR 16.2; CI 3.9–67.8). The presence of polyhydramnios was independently associated with fetuses who had aminoacidopathy disease (OR 4.4; CI 1.4–13.2). The presence of NIHF was independently associated with fetuses who had GSD (OR 74.2; CI 10.3–534).

**TABLE 4:** Multivariate logistic regression—the association between pregnancy complications and newborns with inborn errors of metabolism.

Characteristic	OR^a^	95% CI^a^	*p*-value
Polyhydramnios	2.05	1.14–3.67	**0.016**
Fetal growth restriction	2.36	1.28–4.35	**0.006**
Preterm delivery	1.93	1.30–2.87	**0.001**
Bedouin origin	8.42	4.92–14.4	**<0.001**
HELLP syndrome	1.83	0.80–4.19	0.2
Nonimmune hydrops fetalis	13.9	3.16–61.2	**<0.001**

^a^ OR, odds ratio; CI, confidence interval.

**TABLE 5:** Multivariate logistic regression—the association between clinical risk factors and the delivery of neonates with inborn errors of metabolism.

Characteristic	Preterm birth			Polyhydramnios			HELLP syndrome			Fetal growth restriction		
	OR^a^	95% CI^a^	*p*-value	OR^a^	95% CI^a^	*p*-value	OR^a^	95% CI^a^	*p*-value	OR^a^	95% CI^a^	*p*-value
Neonates with inborn errors of metabolism	2.0	1.4–3	**<0.001**	2.08	1.17–3.71	**0.013**	1.89	0.82–4.33	0.13	2.24	1.2–4.19	**0.012**
Polyhydramnios	1.5	1.43–1.62	**<0.001**	—	—	—	—	—	—	0.81	0.71–0.93	**0.003**
Preterm delivery	—	—	—	1.49	1.40–1.59	**<0.001**	11.0	10.2–11.8	**<0.001**	3.62	3.42–3.82	**<0.001**
Fetal growth restriction	3.6	3.4–3.8	**<0.001**	0.81	0.71–0.93	**0.003**	3.30	2.95–3.69	**<0.001**	—	—	—
HELLP syndrome	11.0	10.2–11.8	**<0.001**	—	—	—	—	—	—	3.31	2.96–3.70	**<0.001**
Bedouin origin	1.0	1.0–1.1	0.2	0.94	0.90–0.98	**0.004**	1.07	0.99–1.15	0.11	1.39	1.32–1.46	**<0.001**
Nonimmune hydrops fetalis	—	—	—	17.9	11.2–28.7	**<0.001**	1.37	0.55–3.43	0.5	—	—	—

^a^ OR, odds ratio; CI, confidence interval.

**TABLE 6:** Multivariate logistic regression—the association between fetal inborn errors of metabolism and pregnancy complications.

Characteristic	Lysosomal storage disease			Mitochondrial disease			Glycogen storage disease			Aminoacidopathy disease		
	OR^a^	95% CI^a^	*p*-value	OR^a^	95% CI^a^	*p*-value	OR^a^	95% CI^a^	*p*-value	OR^a^	95% CI^a^	*p*-value
Preterm delivery	3.41	1.61–7.22	**0.001**	1.7	0.8–3.6	0.2	—	—	—	—	—	—
Fetal growth restriction	3.17	1.06–9.49	**0.039**	4.7	1.9–12.0	**0.001**	—	—	—	—	—	—
HELLP syndrome	—	—	—	5.6	1.8–17.0	**0.003**	—	—	—	—	—	—
Polyhydramnios	—	—	—	—	—	—	2.67	0.83–8.56	0.10	4.4	1.4–13.2	**0.01**
Bedouin origin	39.7	5.38–292	**<0.001**	16.2	3.9–67.8	**<0.001**	2.32	0.88–6.09	0.087	—	—	—
Nonimmune hydrops fetalis	26.4	3.39–206	**0.002**	-	—	—	74.2	10.3–534	**<0.001**	—	—	—

^a^ OR, odds ratio; CI, confidence interval.

## Discussion

The principal findings of our study are as follows: 1) IEM pregnancies are complicated with a higher rate of polyhydramnios, HELLP syndrome, preterm birth, and NIHF; 2) the majority of women with an IEM-affected infant was of Bedouin origin; 3) infants with IEM had a lower mean birthweight, higher rate of low Apgar scores at 1′ and 5′ minutes, FGR, persistent hypoglycemia, metabolic lactic acidosis, hyperammonemia, dilated/hypertrophic cardiomyopathy, postpartum death <28 days, and neonatal death between 29 days and 1 year; 4) IEM neonates as a group were independently associated with preterm birth, polyhydramnios, FGR, and NIHF. Moreover, our study identified that each family of metabolic diseases is independently associated with specific pregnancy complications (i.e., mitochondrial diseases are associated with HELLP syndrome and LSD are associated with NIHF).

Our observation that there are different antenatal presentations of IEM in mothers and their fetuses is novel. For example, polyhydramnios and HELLP syndrome were significantly more common in women with an offspring affected by IEM. Polyhydramnios was previously reported as the first manifestation of some IEM, especially LSD (Kruszka and Regier, 2019), which explains the higher prevalence in pregnancies with affected fetuses in the current study. There are several mechanisms by which different IEMs can cause polyhydramnios: 1) aminoacidopathy, such as NKH, since glycine toxicity resulting from NKH begins *in utero*. The amino acid glycine plays an important role in neurotransmission, and therefore, the accumulation of glycine causes CNS damage. Following severe CNS damage, polyhydramnios can occur in fetuses as a result of decreased swallowing (Sterniste et al., 1998); 2) Bellini et al. (2009) studied different causes of polyhydramnios as part of NIHF and found that the main reasons for polyhydramnios in LSD were anemia or liver failure that lead to low plasma oncotic pressure results in polyhydramnios.

Our finding of the independent association between fetal mitochondrial disease and maternal HELLP syndrome is novel. Previous reports have suggested an association between fetal IEM, such as LCHAD deficiency (Wilcken et al., 1993; Preece and Green, 2002; Journal et al., 2005), and other FAOD of the fetuses (Innes et al., 2000; Matern et al., 2001; Nelson and Alters, 2018) and the development of maternal HELLP syndrome. This observation is explained by the accumulation of 3-hydroxy-fatty acids that may serve as a maternal hepatic toxin or that the cause can be a disruption in FAOD in the placenta (Strauss et al., 1999; Ibdah et al., 2000). There is an association between the development of HELLP syndrome and preeclampsia, with a plausible explanation by Illsinger et al. (2010), describing a possible pathogenesis of these two disorders, thus suggesting that in addition to FAOD, other factors may cause these obstetric complications by affecting mitochondrial function. Mitochondrial dysfunction can lead to abnormal energy production, possibly contributing to the dysfunction of the fetal–placental unit. Free radicals produced due to mitochondrial dysfunctioning may result in the inhibition of nitric oxide released from endothelial cells, leading to vascular changes, therefore resulting in preeclampsia and HELLP syndrome (Slaghekke et al., 2006; Illsinger et al., 2010). Additional support for the association between HELLP syndrome and mitochondrial diseases can be deduced from the study by Vaka et al. (2018), who described the association between reactive oxygen species (ROS) production in mitochondrial dysfunction and the development of preeclampsia by a mechanism of vascular placental ischemia (Raijmakers et al., 2004). Therefore, it can be proposed that the formation of ROS in fetuses with mitochondrial dysfunction may lead to the development of maternal HELLP syndrome.

Fetuses with IEM were found to have an independent risk factor for FGR. FGR was described previously in different types of LSD, such as GM1 gangliosidosis (Roberts et al., 1991a; Brunetti-Pierri et al., 2007). This is an important finding because if FGR is detected during sequential prenatal ultrasounds in such cases, the diagnosis of IEM should be considered and sought out in populations at risk. Indeed, in cases with a high index of suspicion for IEM and the presence of FGR, genetic counseling and amniocentesis should be offered. Postnatally, the diagnosis can also rely on placental examination, which can demonstrate diffuse vacuolar changes (Libbrecht et al., 2020; Wongkittichote et al., 2021).

The association between NIHF and IEM is well described in the literature. Various forms of IEM are expressed clinically in NIHF. In LSD, there are several mechanisms for excessive fluid accumulation resulting in NIHF. It may result from hypoproteinemia due to liver dysfunction (Nicolaides et al., 1985; Staretz-Chacham et al., 2009) and from obstruction of the venous blood flow caused by organomegaly and visceromegaly secondary to accumulating storage material (Hutchison et al., 1982; Staretz-Chacham et al., 2009; Whybra et al., 2012). The prognosis of NIHF is often poor, with a high recurrence rate. For this reason, obtaining an IEM diagnosis is vital for managing an ongoing gestation and assessing the risk of recurrence in future pregnancies. One way to achieve an early diagnosis of IEM is to perform a histologic placental examination whenever there is a suspicion of hydrops at birth or detect by ultrasound, and if cells are highly vacuolated or demonstrate storage, enzyme testing should be performed (Roberts et al., 1991a; Roberts et al., 1991b; Nelson et al., 1993; Hale et al., 1995; Denis et al., 1996; Molyneux et al., 1997; Soma et al., 2000; Groener et al., 2003; Froissart et al., 2005; Vedder et al., 2006; Staretz-Chacham et al., 2009).

The incidence of the IEM that we have described in our study is in accord with that reported by Hazan et al. (2020). Due to the high rate of consanguinity, the Negev region has a substantially higher incidence of IEM than the reported global incidence (Waters et al., 2018). Indeed, the incidence of the three more common groups of IEM disorders that we found in our study are as follows: LSD 14.1 per 100,000, mitochondrial diseases 11.8 per 100,000, and GSD 8.8 per 100,000 were higher than the reported global incidence by Waters et al. (2018) and Adeloye of 13.3 per 100,000 for LSD, 8.2 per 100,000 for mitochondrial diseases, and 6.19 per 100,000 for GSD. In a review of IEM diagnosed in British Columbia (with a predominantly white population), the estimated incidence of LSD was 7.6 per 100,000, of mitochondrial diseases was 3.2 per 100,000, and of GSD was 2.3 per 100,000 (Applegarth et al., 1969–1996). A plausible explanation for the significant differences in the incidence of the diseases would be related to the composition of the populations included in each study. The majority of women included in our study were of Bedouin origin, who are known to have a higher consanguinity rate, as reported previously (Hazan et al., 2020). In their study, Moammar et al. (2010) discussed the incidence of IEM in the Eastern Province of Saudi Arabia, where there is a high consanguinity rate, and families tend to have more children, similar to the Bedouin population in the Negev region of Israel; the incidence of the same three groups of IEM in their study was LSD 44 per 100,000, mitochondrial diseases 8 per 100,000, and GSD 10 per 100,000, which with the exception of LSD incidence, are quite similar to our report.

The clinical implications of our study are that many of the IEMs may be diagnosed during pregnancy, near the time of delivery, or through newborn screening tests (Pourfarzam and Zadhoush, 2013; Kliegman et al., 2016); whenever there is a high index of suspicion for a specific disorder in the fetus with a known family mutation, a prenatal diagnosis should be sought. Prenatal confirmation of IEM in the fetus may influence the management of pregnancy, with specialized treatment consideration offered to the mother during gestation or for the infant immediately after birth, according to the specific diagnosis (Walter, 2000; Illsinger and Das, 2010). Due to newly developed treatments for these rare diseases in recent years, making an earlier diagnosis will highly ameliorate the prognosis.

### Strengths and limitations of the study

This study was conducted as a population-based study because it represents the true incidence of these disorders among the maternity population in Negev. Beyond that, the external validity of the study would be extremely low if we included only women of Bedouin origin. Due to the rarity of diseases, the numbers in the individual IEM groups are very low. Another limitation is that in this study, we have included a large number of healthy women as the control group, in addition to the cases of patients with IEM, although this population is relatively undiversified.

## Conclusion

The novelty of this study in relation to the existing literature is that there is not much information on pregnancy complications in healthy women carrying fetuses with IEM. Most articles dealing with pregnancy complications refer to pregnancies of women who have IEM themselves; therefore, the current study highlights the importance of pregnancy follow-up in healthy women and a high index of suspicion for complications of fetal metabolic diseases in populations at risk. Defects of distinct metabolic pathways may already be of significant relevance *in utero* and for clinical manifestations in the early fetal and neonatal periods. Impaired pathways may influence fetal intrauterine growth and the mother’s health. Production of a toxic or energy-deficient intrauterine environment, modification of the content and function of membranes, or disturbance of the normal expression of intrauterine genes may be responsible for fetal intrauterine injury and developmental disorders. This will allow prenatal diagnosis and personalized targeted treatment (Illsinger and Das, 2010). Early diagnosis of IEM, especially during pregnancy, can significantly improve outcomes, so it is essential to increase clinician awareness globally and ensure access to necessary investigations to avoid missed diagnoses (Levy, 2009).

## Data Availability

The raw data supporting the conclusions of this article will be made available by the authors, without undue reservation.
