# Supplementary material for: Characterization of pregnancy outcome of women with an offspring with inborn errors of metabolism: A population-based study
Source: Front Genet. 2022 Nov 9;13:1030361. doi: 10.3389/fgene.2022.1030361 (PMC9683332; doi:10.3389/fgene.2022.1030361)
Supplement: Supplementary file 1 [file DataSheet1.docx]

Supplementary Material

# Supplementary Tables

**Supplementary Table 1**- The distribution of the types of inborn error of metabolism in the study population.

| **Disease** | **Sub Diagnosis** | **Number of Cases** | **Overall incidence/ 100,000 live births** |
| --- | --- | --- | --- |
| Lysosomal Storage Disease (LSD) | Mucopolysaccharidosis (MPS) type 1H | 1 | 0.30 |
|  | Mucopolysaccharidosis type 1HS | 1 | 0.30 |
|  | Mucopolysaccharidosis type 3A | 7 | 2.07 |
|  | Mucopolysaccharidosis type 3B | 1 | 0.30 |
|  | Mucopolysaccharidosis type 4A | 11 | 3.25 |
|  | Mucopolysaccharidosis type 6 | 2 | 0.59 |
|  | Other Mucopolysaccharidosis | 2 | 0.59 |
|  | Niemann Pick C Disease (NPC) | 16 | 4.72 |
|  | Pompe disease (GSD type 2) | 7 | 2.07 |
|  | **Total:** | **48** | **14.17** |
| Mitochondrial Diseases | Complex 1 deficiency | 11 | 3.25 |
|  | Complex 3 deficiency | 11 | 3.25 |
|  | Complex 4 deficiency | 1 | 0.30 |
|  | Complex 5 deficiency | 7 | 2.07 |
|  | Kearns Sayre syndrome (KSS) | 1 | 0.30 |
|  | Mitochondrial DNA depletion | 2 | 0.59 |
|  | Mitochondrial Neuro-Gastrointestinal Encephalopathy (MNGIE) Disease | 1 | 0.30 |
|  | Pyruvate dehydrogenase (PDH) deficiency type 1A | 1 | 0.30 |
|  | Pyruvate dehydrogenase (PDH) deficiency type E3 | 2 | 0.59 |
|  | Trans-membrane protein 70 (TMEM70) deficiency | 2 | 0.59 |
|  | Other mitochondrial disease | 1 | 0.30 |
|  | **Total:** | **40** | **11.81** |
| Glycogen Storage Disease (GSD) | Glycogen storage disease type 0 | 1 | 0.30 |
|  | Glycogen storage disease type 1A | 1 | 0.30 |
|  | Glycogen storage disease type 1B | 12 | 3.54 |
|  | Glycogen storage disease type 3 | 4 | 1.18 |
|  | Glycogen storage disease type 6 | 8 | 2.36 |
|  | Glycogen storage disease type 9 | 1 | 0.30 |
|  | Other Glycogen storage disease | 3 | 0.89 |
|  | **Total:** | **30** | **8.85** |
| Aminoacidopathy | Maple Syrup Urine Disease (MSUD) | 9 | 2.66 |
|  | Late onset Non-Ketotic Hyperglycinemia (NKH) | 2 | 0.59 |
|  | Non-Ketotic Hyperglycinemia | 13 | 3.84 |
|  | **Total** | **24** | **7.08** |
| Peroxysomal Diseases | Adrenoleukodystrophy (ALD) Disease | 1 | 0.30 |
|  | Zellweger Disease | 16 | 4.72 |
|  | Other Peroxysomal Disease | 1 | 0.30 |
|  | **Total:** | **18** | **5.31** |
| Fatty Acid Oxidation Disease (FAOD) | Carnitine Palmitoyl-Transferase 1A (CPT1A) | 1 | 0.30 |
|  | Carnitine Palmitoyl- Transferase 2 (CPT2) | 2 | 0.59 |
|  | Long Chain 3-Hydroxyl-CoA Dehydrogenase Deficiency (LCHADD) | 1 | 0.30 |
|  | Multiple Acyl-CoA Dehydrogenase Deficiency (MADD) | 2 | 0.59 |
|  | Medium Chain acyl-CoA Dehydrogenase Deficiency (MCADD) | 4 | 1.18 |
|  | Very Long Chain Acyl-CoA Dehydrogenase Deficiency (VLCADD) | 7 | 2.07 |
|  | **Total:** | **17** | **5.02** |
| Organic Acidurias | Glutaric Aciduria (GA) | 7 | 2.07 |
|  | **Total:** | **7** | **2.07** |

*Data is presented as: n(%); Mean ±S.D; Median [Interquartile range].*

**Supplementary Table 2**- Maternal pregnancy outcome, according to the types of Inborn error of metabolism.

|  | **Outcome** | **Lysosomal Storage Disease (48)** | **Mitochondrial Diseases (40)** | **Glycogen Storage Disease (30)** | **Aminoacidopathy (24)** | **Peroxysomal Diseases (18)** | **Fatty Acid Oxidation Disease (17)** | **Organic Acidurias (7)** | **P-Value** |
| --- | --- | --- | --- | --- | --- | --- | --- | --- | --- |
| Antepartum | Polyhydramnios | 2 (4.2) | 1 (2.5) | 3 (10.0) | 3 (12.5) | 3 (16.7) | 1 (5.9) | 0 (0.0) | 0.374 |
|  | Oligohydramnios | 2 (4.2) | 2 (5.0) | 0 (0.0) | 0 (0.0) | 0 (0.0) | 2 (11.8) | 0 (0.0) | 0.315 |
|  | Gestational Diabetes | 2 (4.2) | 2 (5.0) | 0 (0.0) | 1 (4.2) | 0 (0.0) | 2 (11.8) | 0 (0.0) | 0.506 |
|  | Gestational Hypertension | 2 (4.2) | 0 (0.0) | 0 (0.0) | 0 (0.0) | 1 (5.6) | 0 (0.0) | 0 (0.0) | 0.468 |
|  | Preeclampsia | 3 (6.2) | 5 (12.5) | 2 (6.7) | 0 (0.0) | 0 (0.0) | 0 (0.0) | 1 (14.3) | 0.263 |
|  | HELLP Syndrome | 1 (2.1) | 4 (10.0) | 1 (3.3) | 0 (0.0) | 0 (0.0) | 0 (0.0) | 0 (0.0) | 0.224 |
|  | Acute Fatty Liver of Pregnancy | 0 (0.0) | 0 (0.0) | 0 (0.0) | 0 (0.0) | 0 (0.0) | 0 (0.0) | 0 (0.0) | N/A |
|  | Non-Immune Hydrops Fetalis | 1 (2.1) | 0 (0.0) | 1 (3.3) | 0 (0.0) | 0 (0.0) | 0 (0.0) | 0 (0.0) | 0.9 |
| Intrapartum | Preterm Delivery | 15 (31.2) | 9 (22.5) | 5 (16.7) | 4 (16.7) | 5 (27.8) | 0 (0.0) | 0 (0.0) | 0.098 |
|  | Preterm PROM | 7 (14.6) | 3 (7.5) | 4 (13.3) | 4 (16.7) | 3 (16.7) | 2 (11.8) | 0 (0.0) | 0.834 |
|  | Birth Type |  |  |  |  |  |  |  | 0.493 |
|  | Vaginal Delivery | 35 (72.9) | 31 (77.5) | 22 (73.3) | 14 (58.3) | 15 (83.3) | 13 (76.5) | 6 (85.7) |  |
|  | Caesarean Section | 10 (20.8) | 7 (17.5) | 6 (20.0) | 5 (20.8) | 2 (11.1) | 2 (11.8) | 0 (0.0) |  |
|  | Breech Delivery | 0 (0.0) | 1 (2.5) | 0 (0.0) | 1 (4.2) | 0 (0.0) | 0 (0.0) | 0 (0.0) |  |
|  | Mechanical Delivery | 0 (0.0) | 1 (2.5) | 0 (0.0) | 3 (12.5) | 0 (0.0) | 1 (5.9) | 0 (0.0) |  |
|  | Hospitalization Days | 4.51 ±2.07 | 4.65 ±2.12 | 5.25 ±2.17 | 4.35 ±1.58 | 5.24 ±2.86 | 4.76 ±1.92 | 6.00 ±2.10 | 0.425 |
|  | ICU | 0 (0.0) | 1 (2.5) | 0 (0.0) | 0 (0.0) | 0 (0.0) | 0 (0.0) | 0 (0.0) | 0.728 |
| Postpartum | Endometritis | 0 (0.0) | 0 (0.0) | 0 (0.0) | 0 (0.0) | 0 (0.0) | 0 (0.0) | 0 (0.0) | N/A |
|  | Urinary Tract Infection | 0 (0.0) | 0 (0.0) | 0 (0.0) | 0 (0.0) | 0 (0.0) | 2 (11.8) | 0 (0.0) | 0.003 |
|  | Infection | 0 (0.0) | 0 (0.0) | 1 (3.3) | 0 (0.0) | 0 (0.0) | 0 (0.0) | 0 (0.0) | 0.523 |
|  | Fever | 0 (0.0) | 1 (2.5) | 0 (0.0) | 0 (0.0) | 0 (0.0) | 0 (0.0) | 0 (0.0) | 0.756 |

*Data is presented as: n(%); Mean (S.D.); Median [Interquartile range].*

**Supplementary Table 3***-* Fetal and neonatal outcome, according to the types of Inborn error of metabolism.

| **Outcome** | **Lysosomal Storage Disease (48)** | **Mitochondrial Diseases (40)** | **Glycogen Storage Disease (30)** | **Aminoacidopathy (24)** | **Peroxysomal Diseases (18)** | **Fatty Acid Oxidation Disease (17)** | **Organic Acidurias (7)** | **P-Value** |
| --- | --- | --- | --- | --- | --- | --- | --- | --- |
| Gender Infant, |  |  |  |  |  |  |  |  |
| Male | 30 (62.5) | 20 (50.0) | 17 (56.7) | 14 (58.3) | 10 (55.6) | 8 (47.1) | 3 (42.9) | 0.871 |
| Gestational Age at Delivery (weeks) | 263.60 ±19.38 | 268.68 ±15.71 | 270.39 ±14.33 | 261.09 ±31.99 | 275.12 ±17.88 | 274.47 ±7.34 | 275.83 ±8.89 |  |
| Apgar 1 min<5 | 3 (6.3) | 0 (0.0) | 0 (0.0) | 2 (8.4) | 2 (11.1) | 0 (0.0) | 0 (0.0) | 0.008 |
| Apgar 5 min<7 | 3 (6.3) | 0 (0.0) | 0 (0.0) | 1(4.2) | 3 (16.7) | 0 (0.0) | 0 (0.0) | **<0.001** |
| Birthweight (grams) | 2858.15 ±735.12 | 2711.22 ±528.50 | 3227.18 ±572.46 | 2932.17 ±864.59 | 2727.88 ±592.06 | 3195.35 ±478.34 | 3098.50 ±346.40 | **0.016** |
| AGA | 34 (70.8) | 31 (77.5) | 26 (86.7) | 20 (83.3) | 14 (77.8) | 13 (76.5) | 7 (100.0) | 0.128 |
| LGA | 5 (10.4) | 1 (2.5) | 3 (10.0) | 4 (16.7) | 0 (0.0) | 2 (11.8) | 0 (0.0) |  |
| SGA | 9 (18.8) | 8 (20.0) | 1 (3.3) | 0 (0.0) | 4 (22.2) | 2 (11.8) | 0 (0.0) |  |
| Neonatal ICU Admission | 16 (33.3) | 18 (45.0) | 17 (56.7) | 14 (58.3) | 16 (88.9) | 10 (58.8) | 1 (14.3) | **0.001** |
| Hospitalization Days | 9.96 ±16.54 | 7.62 ±10.39 | 13.59 ±18.73 | 6.12 ±5.57 | 34.83 ±34.76 | 11.47 ±9.97 | 5.17 ±2.14 | **<0.001** |
| Fetal Growth Restriction | 5 (10.4) | 6 (15.0) | 0 (0.0) | 0 (0.0) | 1 (5.6) | 1 (5.9) | 0 (0.0) | 0.153 |
| Splenomegaly | 0 (0.0) | 0 (0.0) | 0 (0.0) | 0 (0.0) | 0 (0.0) | 0 (0.0) | 0 (0.0) | N/A |
| Hepatomegaly | 0 (0.0) | 0 (0.0) | 0 (0.0) | 0 (0.0) | 0 (0.0) | 0 (0.0) | 0 (0.0) | N/A |
| Ascites | 0 (0.0) | 0 (0.0) | 0 (0.0) | 0 (0.0) | 0 (0.0) | 0 (0.0) | 0 (0.0) | N/A |
| Antepartum Death | 0 (0.0) | 1 (2.5) | 0 (0.0) | 0 (0.0) | 0 (0.0) | 0 (0.0) | 0 (0.0) | 0.728 |
| Asphyxia | 0 (0.0) | 1 (2.5) | 0 (0.0) | 0 (0.0) | 1 (5.6) | 0 (0.0) | 0 (0.0) | 0.485 |
| Meconium Aspiration | 0 (0.0) | 0 (0.0) | 0 (0.0) | 0 (0.0) | 0 (0.0) | 0 (0.0) | 0 (0.0) | N/A |
| Hemorrhage | 0 (0.0) | 0 (0.0) | 0 (0.0) | 1 (4.2) | 0 (0.0) | 1 (5.9) | 0 (0.0) | 0.292 |
| Shoulder Dystocia | 0 (0.0) | 0 (0.0) | 0 (0.0) | 1 (4.2) | 0 (0.0) | 0 (0.0) | 0 (0.0) | 0.349 |
| Persistent Hypoglycemia | 3 (6.2) | 2 (5.0) | 7 (23.3) | 2 (8.3) | 0 (0.0) | 4 (23.5) | 0 (0.0) | 0.026 |
| Metabolic Lactic Acidosis | 0 (0.0) | 0 (0.0) | 1 (3.3) | 1 (4.2) | 0 (0.0) | 0 (0.0) | 1 (14.3) | 0.104 |
| Hyperammonemia | 0 (0.0) | 0 (0.0) | 0 (0.0) | 1 (4.2) | 0 (0.0) | 0 (0.0) | 0 (0.0) | 0.349 |
| Hypotonia | 9 (18.8) | 11 (27.5) | 3 (10.0) | 5 (20.8) | 1 (5.6) | 1 (5.9) | 0 (0.0) | 0.154 |
| Developmental Delay | 12 (25.0) | 19 (47.5) | 5 (16.7) | 1 (4.2) | 4 (22.2) | 0 (0.0) | 2 (28.6) | 0.001 |
| Dilated/ Hypertrophic Cardiomyopathy | 7 (14.6) | 10 (25.0) | 0 (0.0) | 0 (0.0) | 0 (0.0) | 0 (0.0) | 0 (0.0) | **0.001** |
| Seizures | 0 (0.0) | 0 (0.0) | 0 (0.0) | 0 (0.0) | 1 (5.6) | 0 (0.0) | 0 (0.0) | 0.159 |
| Postpartum Death (<28 Days) | 1 (2.1) | 5 (12.5) | 0 (0.0) | 11 (45.8) | 3 (16.7) | 2 (11.8) | 0 (0.0) | **<0.001** |
| Neonatal Death (Between 29 Days- 1 Year) | 6 (12.5) | 5 (12.5) | 1 (3.3) | 2 (8.3) | 12 (66.7) | 1 (5.9) | 0 (0.0) | **<0.001** |

*Data is presented as: n(%); Mean (S.D.); Median [Interquartile range].*
